# Oxidative Potential in Exhaled Air (OPEA) as a Tool for Predicting Certain Respiratory Disorders in the General Adult Population: Cross-Sectional Analysis Nested in the Swiss Health Study

**DOI:** 10.3390/antiox11102079

**Published:** 2022-10-21

**Authors:** Irina Guseva Canu, Maud Hemmendinger, Antonio Toto, Pascal Wild, Caroline Veys-Takeuchi, Murielle Bochud, Guillaume Suárez

**Affiliations:** 1 Department of Occupational and Environmental Health, Center for Primary Care and Public Health (Unisanté), University of Lausanne, 1066 Epalinges, Switzerland; 2 Department of Epidemiology and Health Systems, Center for Primary Care and Public Health (Unisanté), University of Lausanne, 1015 Lausanne, Switzerland

**Keywords:** oxidative stress, reference interval, COPD, SARS-CoV-2

## Abstract

In a pilot clinical study, OPEA allowed for distinguishing participants with and without chronic obstructive pulmonary disease. This study aimed to assess whether abnormal spirometry parameters and immunity against SARS-CoV-2 are associated with increased OPEA and estimating the OPEA reference interval. Swiss adult residents of the Vaud Canton aged 20–69 years randomly selected from the Federal Statistical Office’s registries, speaking French or German, were included and examined between 1 October 2020 and 31 December 2021. General health status and presence of respiratory diseases were assessed by questionnaire and spirometry. Spirometric results were compared with the predicted values and their lower limits of norms of the Global Lung Function Initiative. SARS-CoV-2-seroprevalence was assessed using the Luminex-based test of IgG. Statistical analysis consisted of unilateral t-tests and ANOVA. Lower and upper limit of OPEA reference interval with associated 90%-confidence interval (90%CI) were estimated for the sub-sample of healthy adults by bootstrap, after excluding outliers. The study sample included 247 participants. SARS-CoV-2-seropositive participants and those with an obstructive syndrome had a significantly higher OPEA than seronegative and healthy participants. The estimated reference interval was: −0.0516 (90%CI = −0.0735; −0.0316); −0.0044 (90%CI = −0.0224; 0.0153). OPEA could predict inflammatory-based respiratory disorders, but needs further validation in different settings and for other pathologies.

## 1. Introduction

**Figure 1:**
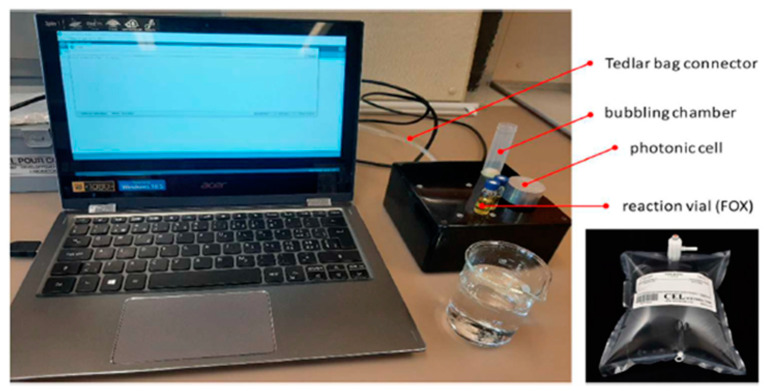
Experimental setup for the determination of OPEA. The setup comprises the OPEA analyzer (black box) and its different computer-driven mechanical/optical components. Disposable Tedlar bag (bottom right) was used for air sample collection (1 L).

Oxidative stress is an important indicator in the toxicological pathways, reflecting the expression of almost all diseases [1]. Oxidative stress results from a redox imbalance between the production of reactive oxygen species (ROS), which are mainly free radicals and peroxides, and the antioxidant defenses of an organism [2,3]. Excessive production of ROS results in oxidative damage to proteins, lipids, free amino acids, and DNA and causes structural and functional cellular changes [1]. In the oxidative stress paradigm, the determination of the oxidative potential in the exhaled air (OPEA) is a novel and promising approach to detect respiratory disorders such as chronic obstructive pulmonary disease (COPD), for which oxidative stress and inflammation are the likely physio-pathological mechanism. A photonic-based OPEA analyzer enables the non-invasive determination of the OPEA by sampling 1 L of exhaled air in a disposable Tedlar bag (Figure 1).

**Figure 2:**
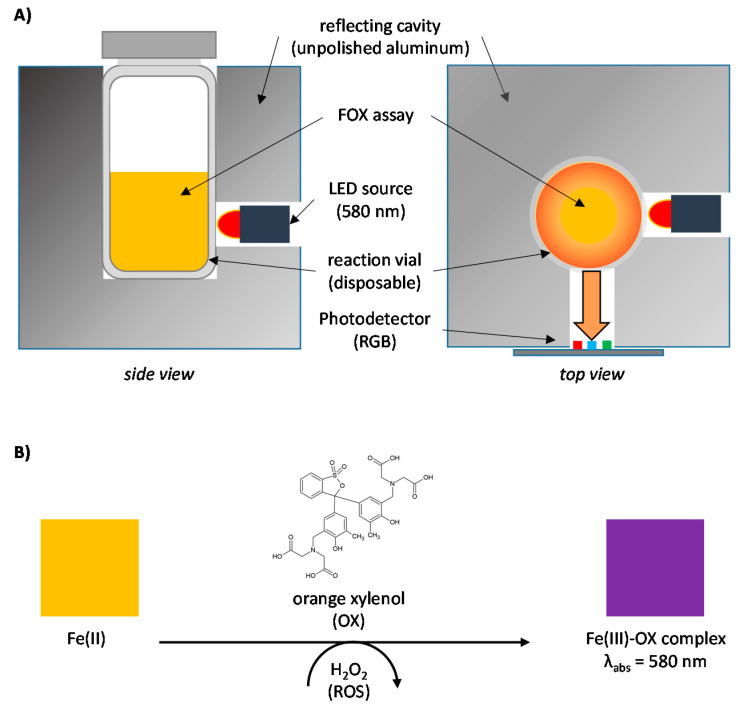
Principles of OPEA detection. (**A**) Schematic representation of the photonic detection strategy based on multiscattering-enhanced absorbance that enables highly sensitive absorbance change detection. (**B**) Description of the FOX assay in which the formation of a purple Fe(III)–OX complex reflects the overall redox balance versus Fe(III)/Fe(II).

The OPEA analyzer relies on a highly sensitive detection strategy, the so-called multiscattering-enhanced absorbance (MEA), which is particularly suitable to determine the overall oxidative potential in extremely diluted samples (EU and U.S. granted patents) [4,5]. The detection principle is based on the amplification of the measured absorbance due to the multiscattering regime occurring when light propagates through an optically random medium (heterogeneity of refractive indices) or a reflecting material. As a cheap and efficient way to achieve absorbance enhancement, the optical cell of the OPEA analyzer consists of an unpolished aluminum cavity exhibiting strong reflection behavior in which both the light source (LED 580 nm) and the photodetector (RGB CMOS sensor) are positioned with an angle of 90° (Figure 2A). Using this simple MEA strategy, the photonic cell enables improving the optical path length and increasing the average time-lag of a photon in the system. The colorimetric assay adapted to this optical detection instrument—namely FOX (Fe-Orange xylenol)—is based on the formation of the purple Fe(III)–orange xylenol complex (absorption at 580 nm) as soon as Fe(II) is oxidized in the presence of oxidative species, in particular hydrogen peroxide (H_2_O_2_). In the course of the reaction, the initial solution progressively turns from yellow to purple as a sign of the oxidation reaction taking place (Figure 2B). In brief, FOX solution is placed in glass vials (1 mL) in which the sample (300 µL) is added prior to analysis. Once placed in the optical cell cavity, all oxidants in the sample are quantitatively determined as the measured absorbance (580 nm) varies linearly with the amount of H_2_O_2_ equivalents (pmol). The calibration of the OPEA analyzer with H_2_O_2_ shows the efficiency of the sensing strategy with an extremely low limit-of-detection achieved below 3 pmol compared to a LOD > 200 pmol with the standard cuvette-spectrometer configuration [6].

To evaluate the OPEA analyzer and OPEA significance with respect to chronic obstructive pulmonary disease (COPD), a pilot clinical study was previously conducted in France at the Department of Pneumology and Occupational Pathology of an Intercommunal Hospital Center in Créteil. This study included 53 COPD and healthy patients and showed that OPEA allows discriminating patients with and without COPD, particularly in non-smokers [6]. Moreover, a statistically significant correlation was found (Pearson coefficient: −0.33) between the calculated OPEA and the FEV1/FVC ratio, which is decreased in COPD patients, indicating the degree of bronchiolar obstruction [6].

These results suggest that OPEA is a promising candidate biomarker for predicting lung diseases, which can be used non-invasively. However, to validate it as a clinical biomarker, several further steps should be accomplished. On the one hand, the described OPEA measurement should be fully characterized in the controlled laboratory environment, then implemented in different situations and on larger study samples, and finally described statistically, providing its reference interval [7].

The objectives of this study were twofold. First, we aimed to assess whether positive SARS-CoV-2 serology and/or abnormal lung function parameters were associated with increased OPEA in a representative sample of the general adult population. Second, we aimed to calculate the OPEA reference interval in a sample restricted to healthy adults. The population-based reference interval is the most widely used tool for the interpretation of individual laboratory test results [8,9]. Therefore, providing the reference interval for OPEA is critical for further use of this biomarker in research and clinical practice.

## 2. Materials and Methods

### 2.1. Study Design

This study was nested within the pilot phase of a national project called the “Swiss Health Study” (SHeS) coordinated by the Swiss Federal Office of Public Health in collaboration with Unisanté [10]. The aim of the pilot phase was twofold. The fist aim wasto assess the feasibility of a national initiative while testing harmonized procedures and infrastructures. The second aim was to assess trends in the relationship between exposure to environmental substances and human health and in the distribution and prevalence of important diseases in Switzerland. The long-term objective of the project is to establish an epidemiological cohort study of 100,000 adults with the following three purposes: (1) To gather high quality health and exposure data and create a large national biobank with the collected data; (2) to set reference exposure values for the Swiss population; and (3) to support health policy decisions. Given the setting, the aims and the infrastructure developed for SHeS, it represented a great opportunity to nest the OPEA study within this cohort. Indeed, it enabled us to use the direct a priori sampling approach, where exclusion criteria were applied before biological sample collection.

### 2.2. Study Sample

The SHeS involved Swiss residents in the Berne and Vaud Counties, aged 20 to 69. According to the protocol, the Swiss Federal Office of Statistics (FSO) randomly selected participants from the county population registries. To recruit participants, the written invitation send by postal service was the main channel. Study invitation letters were sent out in several recruitment waves to participants selected by the FSO. These letters contained a cover letter, the study information, and the study flyer. The study information informed the participants about the aims, procedures, assessments, and investigators involved in the pilot study; the flyer is a short version of this. Prospective participants not responding to the invitation letter were re-contacted with 1–2 reminder letters. Furthermore, snowball advertisements in organic baskets and at a conference on veganism were used to increase the participation of vegetarians and vegans, as this population represented a very small fraction of the FSO selected participant sample. Study participation was voluntary-based and all participants provided written consent before inclusion in the study.

For logistical reasons, this study was restricted to SHeS participants residing in Vaud County. Indeed, the OPEA has to be measured immediately after the sampling participant’s exhaled air and ambient air in a laboratory situated in the Unisanté premises. Participants were invited to the Unisanté study center to provide other biological samples (i.e., blood and urine), undergo a health examination and answer questionnaires related to relevant exposure sources such as nutrition, lifestyle habits, or occupation.

The exclusion criteria were as followed: (1) Not being capable of understanding the study information (e.g., language, psycho-cognitive impairment etc.); (2) unable to respond to the questions (e.g., language, psycho-cognitive or motor impairment etc.); (3) institutionalized persons (e.g., in prisons, nursing homes, etc.); (4) not a Swiss national residing in Switzerland and not a resident according to the definition: B/C/L/F/N or FDFA’s permit holders (minimum permit duration of 12 months); (5) unable to participate in most examinations for medical reasons; (6) unable to attend the visit at the study center for medical reasons; and (7) the unavailability of OPEA measurement.

### 2.3. Data Collection and Management

Study data were collected and managed using the Research Electronic Data Capture (REDCap) web-based software [11]. The advantages of REDCap includes its intuitive interface for validated data entry, audit trails for tracking data manipulation and export procedures, automated export procedures for unified data downloads to the most common statistical packages, and procedures for importing data from external sources [12,13]. The latter is particularly useful for importing the results of the pulmonary functional test and of the laboratory measurements.

Via a self-administered electronic questionnaire, we collected data regarding the participants’ sociodemographic characteristics, occupation, health status, treatment received, life style habits, occupational history, and occupational and environmental exposures. A binary health status was defined using these data as follows. If the participant declared the presence of a respiratory disease (e.g., asthma, chronic bronchitis, COPD, or emphysema) or classified their general health as bad, he/she was classified as unhealthy. Otherwise, he/she was classified as healthy. Self-reported respiratory disorders were compared with the results of the physiological measurements (performed at Unisanté study center) and lab analyses.

### 2.4. Pulmonary Functional Test

Pulmonary function tests were performed by a research nurse, who was trained by a pulmonologist at the Vaud University hospital center. For the last six months of the study, two additional nurses were hired and trained in the same way due to the increasing number of participants recruited. All tests were performed with the EasyOne Air spirometer (ndd Medizintechnik AG, Zürich, Switzerland) which is considered as a reference device due to its similarity in flow sensor technology and its history of clinical application [14,15,16]. The tests were performed at least three times and a maximum of eight times per participant, depending on the quality and reproducibility of the test results. The quality of the measurement was then controlled based on an automatic result displayed by the EasyOne Air spirometer with a gradation of the quality of the test in terms of the result reproducibility.

Pulmonary function test or spirometry allows us to measure several functional parameters enabling the diagnosis of obstructive or restrictive syndromes and the assessment of their severities. These parameters include the Forced Expiratory Volume in 1 s (FEV1), the Forced Vital Capacity (FVC), the Forced Expiratory Fluxes at 25% and 75% of FVC (FEF25–75%), and the FEV1/FVC ratio. According to the Global Initiative for Chronic Obstructive Lung Disease (GOLD) criteria [17], the post-bronchodilation FEV1/FVC ratio is related to the severity of obstructive syndrome. As bronchodilator administration was not possible in the frame of this study, we used standardized equations of the Global Lung Initiative (GLI) [18]. By applying these equations to the observed spirometry values accounting for the participants’ ethnicity, height, age and sex using the R-library rspiro [19], we obtained the predicted values of FEV1, FVC, FEV1/FVC, and FEF25–75%. Then, for each participant and for each parameter, we generated the lower limit of normal (LLN) of the GLI [18]. The comparison of the observed value with the LLN based on a dichotomous variable for each parameter (<LLN versus ≥LLN) allowed us to determine if the participant had an abnormally low value of the spirometric parameter considered (belonging to the lower 5% of the GLI reference distribution).

### 2.5. SARS-CoV-2 Serology Test

As the study was conducted during the coronavirus disease 2019 (COVID-19) pandemic, due to the transmission of severe acute respiratory syndrome coronavirus type 2 (SARS-CoV-2), the presence of SARS-CoV-2 antibodies was chosen as a second respiratory health outcome. SARS-CoV-2 immunoglobulin G (IgG) antibodies become detectable in most cases around 6–15 days after symptom onset [20]. Sera extracted from the participants’ venous blood were analyzed using SenASTrIS (Sensitive Anti-SARS-CoV-2 Spike Trimer Immunoglobulin Serological), a Luminex binding assay [21]. The assay measures the binding of IgG antibodies to the trimeric SARS-CoV-2 S-protein. The overall test result was counted as positive when either the SARS-CoV-2-IgG signal was above the cutoff. Indeterminate test results, meaning a signal just below the predefined cutoff, were retested, and when confirmed, “indeterminate” was counted as negative. The test has a high specificity (99.7%) and sensitivity (96.6%) and has been validated in samples of the general population as well as specific subgroups of people [20].

### 2.6. OPEA Measurement

Exhaled air samples were analyzed directly after sample collection by Unisanté chemists (MH, AT). Since background contaminants present in the ambient air (e.g., particles, NOx, ozone) may influence the oxidative potential in the airways, oxidative potential in the ambient air was measured along with the OPEA and used for normalizing OPEA. All oxidative potential measurements were performed in triplicate. Test results for OPEA were retrieved and transformed as follows. Raw data originating from the photosensor—time intervals, μs—were collected and processed on the OPEA analyzer by a microcontroller board (ATmega328, Arduino, Italy). In real-time, the data are sent from the controller board and compiled in a .txt file on PC (Coolterm software, http://freeware.the-meiers.org/, accessed on 30 August 2022). In brief, every 2 s and for a total time analysis of 180 s, a data line is generated with the values of elapsed time from t0 (s) associated with the normalized absorption index that reflects the FOX reaction advancement. For each OPEA analysis, a unique self-incremented id number is created on the file. The main data treatment consists of calculating the slopes—60 to 150 s interval—for each id and the corresponding correlation coefficient (R^2^). In order to make this process fast and robust, a specific solution was built up with Splunk^®^ software (san Francisco, CA, USA), which classifies the data from Arduino .txt files, generates a graph for visual check of the curves (time–evolution of absorption), and calculates the values of the slopes and R2.

In the generated .xls file, the oxidative potential values are calculated by converting the slope values (s^−1^) into pmol/L_air_ via the linear calibration relationship. Finally, the database compiles the values obtained for the different id measurements and enables the calculation of OPEA and oxidative potential in the ambient air. The OPEA is then divided by oxidative potential in the ambient air as a ratio of OPEA to oxidative potential in the corresponding ambient air. Since both are measured in the same unit (i.e., pmol/L), the resulting value is dimensionless. As OPEA is usually right-skewed, a log_10_ transformation of the ratio allows us to treat it as a normally-distributed variable and use parametric statistical tests.

### 2.7. Statistical Analysis

First, we examined the associations between OPEA and the self-declared and measured health outcomes using univariate analysis. Since the research hypothesis was that the OPEA mean value will be higher in abnormal respiratory health conditions, we used a unilateral *t*-test. To assess the effect of the participants’ characteristics on the OPEA and decide upon the need for multivariate analysis and partitioning of the reference interval, we used the *t*-test for dichotomous variables and the analysis of variance (ANOVA) for other variables. The examined variables were the participants’ sex, age (in five categories: 20–29; 30–39; 40–49; 50–59; 60–71), BMI, diet (omnivore; vegetarian; vegan), and smoking status (non-smokers; current smokers; ex-smokers).

To compute the reference interval, we followed the C28-A3 guideline “Defining, Establishing, and Verifying Reference Intervals in the Clinical Laboratory”, co-produced by the Clinical and Laboratory Standards Institute (CLSI) and the International Federation of Clinical Chemistry (IFCC) [22]. We excluded the participants classified as unhealthy based on the self-declared respiratory diseases and/or bad health. The remaining participants constituted the reference sample. The OPEA distribution and the presence of outliers (i.e., results that were not supposed to belong to the reference population) were examined visually [23]. Outliers were managed using the Tukey method [24]. Finally, the reference interval was calculated as the mean ± 1.96 standard deviation (SD), which corresponds to the central 95% limits or lower limit (LL) and upper limit (UL). For improved precision of the reference interval, the bootstrap method through 50-times resampling of the dataset was applied to obtain smoothed lower and upper limits (LL, UL), and the mean of the reference interval [25]. This resampling procedure was also used to predict the 90% confidence intervals (CI) for the limits of the reference interval.

## 3. Results

The study sample included 247 participants, all Caucasians. Overall, 221 participants were classified as healthy based on their self-declared data (Table 1). Among the unhealthy participants, 15 reported respiratory diseases, most of which were corroborated by spirometry results (data not shown). The remaining participants classified their general health as bad or reported having other diseases. Spirometry results were available for 238 participants (96.36%). The prevalence of abnormal spirometry parameters was less than 9%. Ninety percent of participants performed a SARS-CoV-2 serology test, and 20.65% of them were seropositive. It worth mentioning that among the 51 seropositive participants, 13 were vaccinated against SARS-CoV-2.

Table 2 summarizes the average OPEA value according to the participants’ socio-demographic and lifestyle characteristics. The OPEA values appeared stable across sex and age groups, and across all categories of self-declared smoking and health status. Regarding BMI and diet, however, we observed that obese individuals (BMI > 30) and vegetarians had higher OPEA values (0.03 and 0.02, respectively) than the other categories, although this difference was not statistically significant (*p* = 0.24 and *p* = 0.15, respectively (Table 2)). None of the socio-demographic and lifestyle characteristics considered were associated with OPEA. In contrast, among the health outcomes considered, the positive SARS-CoV-2 serology and FEV1/FVC ratio below LLN (GLI) were associated with a higher OPEA level. As the OPEA appeared invariable with respect to the individual and lifestyle characteristics, no multivariate analysis adjusted for any of them was necessary. Similarly, there was no need to partition the reference interval according to these variables.

After excluding unhealthy participants, the remaining sample consisted of 221 participants. One was identified as an outlier in the OPEA distribution and discarded. Thus, the reference sample included 220 participants. The estimated reference values are summarized in Table 3.

## 4. Discussion

In this study, we addressed two research objectives. First, we confirmed our research hypothesis that OPEA increases in certain respiratory conditions and can be considered as a useful, non-invasive biomarker. We found that OPEA was significantly higher among adults who had experienced a SARS-CoV-2 infection compared to SARS-CoV-2 seronegative adults on one hand and among adults with an obstructive syndrome compared to the adults with normal FEV1/FVC value on the other. These findings confirm the results of the pilot clinical study, where OPEA was found to discriminate the COPD patients from those free of COPD [6], while a clinical COPD diagnosis was based on the GOLD criteria [17]. Furthermore, we found that OPEA is stable with respect to individual characteristics such as sex and age, but also with respect to some exposures such as tobacco smoking or different types of diets. Such a stability was also observed in the pilot clinical study [6] and in two ongoing studies [26,27], and is a valuable characteristic for a candidate biomarker, facilitating its interpretation and therefore its use in clinical practice.

Reliable and accurate reference intervals for laboratory analyses are an integral part of the process of the correct interpretation of clinical laboratory test results [8]. Reference intervals help the clinician in interpreting the test results and are complementary to clinical decision limits. Indeed, while the latter are associated with a significantly higher risk of adverse clinical outcomes or are diagnostic for the presence of a specific disease, the reference intervals describe the typical distribution of results characteristic of a healthy reference population [28]. In establishing the OPEA reference interval, we followed the C28-A3 guideline, which defines all the necessary steps in establishing reference intervals including the selection of reference individuals, pre-analytical and analytical considerations, and estimation of the reference values and the reference interval [22]. We determined the reference interval using direct methods based on a healthy population selected by a priori direct sampling, which is the primary recommendation of the IFCC [8]. Our sample was established using a randomized selection performed by the FSO. The overall participation rate among the FSO selected individuals was 17.5% for answering the questionnaires and 13.7% for both answering the questionnaires and participating in the visit. Although lower than the participation rates in the large Swiss cohorts of general population constituted in the nineties [29,30], the participation rate observed in the present study is comparable with those observed in the more recent cohort studies of general population in Switzerland and elsewhere [31,32]. For instance, in the French CONSTANCES cohort, the participation rate at the enrolment in the cohort was 7.3% [31]. The pandemic context and quite an intensive participant burden are the likely reasons of a lower participation willingness.

The study sample included 247 (73.5%) participants out of 336 participants in the SHeS for whom the OPEA has been measured. In fact, this study started after the pilot phase of SHeS had been launched and reopened after the first wave of SARS-CoV-2 and the corresponding lockdown, and the participants recruited and examined before were not contacted for OPEA measurement. Similarly, the SARS-CoV-2 serology was not performed at the start of the study, which is the reason why this variable had 9.31% of missing data for this variable. Nevertheless, given that the minimal sample size for establishing the reference interval is 120 individuals [33], with 220 participants, we had an adequate sample for achieving our second objective.

We believe that this sample is free of selection bias, even if all participants included were Caucasians and 60% of them were women. Given the demography of the Vaud County residents [34] and the inclusion criteria applied on one hand and a greater willingness to participate in such studies among women on the other hand, this was expected. Consequently, we could not investigate the effect of ethnicity on OPEA in this study sample. Nevertheless, the results from the French–Swiss study of subway workers [26,35] and those from the European cohort of nanotechnology workers [27] showed that OPEA did not differ across ethnicity groups. The tests were not statistically significant, with *p* = 0.158, and *p* = 0.776, respectively (unpublished results). The representativeness of the general healthy population of Vaud County residents should be further confirmed based on the comprehensive analysis of the future national SHeS study. Such an analysis was not possible in the frame of the present pilot study. Nevertheless, some data suggest that the study sample is likely to be representative such as the proportion of individuals with asthma and COPD, which is consistent with the prevalence of these diseases in the general population [36]. This is also true for the proportion of vaccinated participants, which was 56.68% in our sample overall, and 9.29% among those with positive SARS-CoV-2 serology.

Among the limitations, it is worth mentioning that the spirometry results were not validated by a pulmonologist and the lung functional test was not repeated after the administration of a bronchodilator, as recommended by the clinical guidelines enabling asthma or COPD diagnosis. The selection of healthy participants for the reference interval estimation was based on the self-declared data, which could be less precise than a clinical diagnosis. Moreover, as the study was focused on respiratory diseases, we did not examine the effect of other health conditions such as hypertension or diabetes mellitus type 2, and only excluded participants who classified their general health as bad. Although we do not expect any serious bias due to this choice, it should be confirmed in a future study using a more robust disease classification based on diagnosed diseases.

## 5. Conclusions

In the present study, we estimated, for the first time, the OPEA reference interval using a randomly selected sample of the healthy adults residing in Vaud County, Switzerland. The OPEA was found to be significantly higher among SARS-CoV-seropositive participants and those with an obstructive syndrome compared to the seronegative and healthy participants. The OPEA could thus predict some respiratory disorders. However, the results should be confirmed in further studies and different settings. Observational field studies, along with controlled human exposure studies, should enable us to assess the potential effect of occupational and environmental airborne pollutants on the OPEA and how precise the OPEA classification is as an effect versus an exposure biomarker.

## Figures and Tables

**Table 1:** Description of the study sample (N = 247).

Sociodemographic Characteristics	n	%	Health Characteristics	n	%
Sex			Self-declared health status		
Female	150	60.73	Healthy	221	89.47
Male	97	39.27	Unhealthy	26	10.53
Age category (years)			SARS-CoV-2 serology		
20–29	53	21.46	Positive	51	20.65
30–39	48	19.43	Negative	173	70.04
40–49	43	17.41	Unknown	23	9.31
50–59	56	22.67	FEV1		
60–71	47	19.03	Normal	217	87.85
Diet status			<LLN(GLI)	21	8.5
Omnivor	182	73.68	Unknown	9	3.64
Vegetarian	33	13.36	FVC		
Vegan	29	11.74	Normal	231	93.52
Unknown	3	1.21	<LLN(GLI)	7	2.83
Smoking status			Unknown	9	3.64
Non-smoker	134	54.25	FEV1/FVC		
Smoker	28	11.34	Normal	225	91.09
Ex-smoker	85	34.41	<LLN(GLI)	13	5.26
BMI			Unknown	9	3.64
≤25	139	56.28	FEF25-75		
25–30	73	29.55	Normal	220	89.07
≥30	24	9.72	<LLN(GLI)	18	7.29
Unknown	11	4.45	Unknown	9	3.64

**Table 2:** The observed OPEA mean and association with the participants’ characteristics (N = 247).

Characteristic	Category	Observed Mean	95% Conf.	Interval	*p*-Value
Sex	Female	−0.0390	−0.0730	−0.0051	0.51
	Male	−0.0399	−0.0821	0.0023	
Age (y)	20–29	−0.0289	−0.0862	0.0284	0.93
	30–39	−0.0514	−0.1117	0.0089	
	40–49	−0.0279	−0.0916	0.0358	
	50–59	−0.0564	−0.1122	−0.0006	
	60–71	−0.0290	−0.0899	0.0319	
Diet	Omnivore	−0.0451	−0.0759	−0.0143	0.15
	Vegetarian	0.0237	−0.0487	0.0960	
	Vegan	−0.0721	−0.1493	0.0050	
BMI	≤25	−0.0409	−0.0753	−0.0065	0.24
	25–30	−0.0482	−0.0957	−0.0007	
	≥30	0.0315	−0.0514	0.1143	
Smoking	Non-smoker	−0.0274	−0.0633	0.0085	0.62
	Smoker	−0.0587	−0.1372	0.0199	
	Ex-smoker	−0.0518	−0.0969	−0.0068	
Self-declared health	Healthy	−0.0349	−0.0629	−0.0070	0.83
	Unhealthy	−0.0768	−0.1582	0.0046	
SARS-CoV-2 serology	Negative	−0.0561	−0.0875	−0.0247	0.03
	Positive	0.0096	−0.0483	0.0674	
FEV1	Normal	−0.0372	−0.0649	−0.0094	0.23
	<LLN(GLI)	−0.0027	−0.0920	0.0867	
FVC	Normal	−0.0331	−0.0601	−0.0062	0.66
	<LLN(GLI)	−0.0666	−0.2214	0.0883	
FEV1/FVC	Normal	−0.0400	−0.0671	−0.0128	0.04
	<LLN(GLI)	0.0671	−0.0458	0.1800	
FEF25–75	Normal	−0.0374	−0.0650	−0.0098	0.20
	<LLN(GLI)	0.0064	−0.0900	0.1029	

**Table 3:** The OPEA reference distribution and interval in the SHeS sample of healthy adults (N = 220).

Estimated Statistics	OPEA
Mean	−0.0280414
Standard deviation	0.0120425
Lower level	−0.0516443
90%-IC	[−0.0734537; −0.0316189]
Upper level	−0.0044385
90%-IC	[−0.0224059; 0.0153129]

OPEA is normalized as log10 (oxidative potential in exhaled air/oxidative potential in ambient air).

## Data Availability

The data are not publicly available due to ethical and privacy restrictions.
